# A36-dependent Actin Filament Nucleation Promotes Release of Vaccinia Virus

**DOI:** 10.1371/journal.ppat.1003239

**Published:** 2013-03-21

**Authors:** Jacquelyn Horsington, Helena Lynn, Lynne Turnbull, Delfine Cheng, Filip Braet, Russell J. Diefenbach, Cynthia B. Whitchurch, Guna Karupiah, Timothy P. Newsome

**Affiliations:** 1 School of Molecular Bioscience, University of Sydney, Sydney, New South Wales, Australia; 2 The ithree institute, University of Technology Sydney, Sydney, New South Wales, Australia; 3 School of Medical Sciences (Discipline of Anatomy and Histology), The Bosch Institute, The University of Sydney, New South Wales, Australia; 4 Australian Centre for Microscopy & Microanalysis, University of Sydney, Sydney, New South Wales, Australia; 5 Centre for Virus Research, Westmead Millennium Institute, University of Sydney, Westmead, New South Wales, Australia; 6 John Curtin School of Medical Research, Australian National University, Canberra, Australian Capital Territory, Australia; University of Florida, United States of America

## Abstract

Cell-to-cell transmission of vaccinia virus can be mediated by enveloped virions that remain attached to the outer surface of the cell or those released into the medium. During egress, the outer membrane of the double-enveloped virus fuses with the plasma membrane leaving extracellular virus attached to the cell surface via viral envelope proteins. Here we report that F-actin nucleation by the viral protein A36 promotes the disengagement of virus attachment and release of enveloped virus. Cells infected with the A36^YdF^ virus, which has mutations at two critical tyrosine residues abrogating localised actin nucleation, displayed a 10-fold reduction in virus release. We examined A36^YdF^ infected cells by transmission electron microscopy and observed that during release, virus appeared trapped in small invaginations at the plasma membrane. To further characterise the mechanism by which actin nucleation drives the dissociation of enveloped virus from the cell surface, we examined recombinant viruses by super-resolution microscopy. Fluorescently-tagged A36 was visualised at sub-viral resolution to image cell-virus attachment in mutant and parental backgrounds. We confirmed that A36^YdF^ extracellular virus remained closely associated to the plasma membrane in small membrane pits. Virus-induced actin nucleation reduced the extent of association, thereby promoting the untethering of virus from the cell surface. Virus release can be enhanced via a point mutation in the luminal region of B5 (P189S), another virus envelope protein. We found that the B5^P189S^ mutation led to reduced contact between extracellular virus and the host membrane during release, even in the absence of virus-induced actin nucleation. Our results posit that during release virus is tightly tethered to the host cell through interactions mediated by viral envelope proteins. Untethering of virus into the surrounding extracellular space requires these interactions be relieved, either through the force of actin nucleation or by mutations in luminal proteins that weaken these interactions.

## Author Summary

Traversing the plasma membrane of the host cell is a significant challenge for many viruses during the infection cycle, and the efficiency of detachment from the host cell and subsequent release can have implications in pathogenesis. Vaccinia virus exits cells through the loss of an outer membrane but remains attached via viral envelope proteins that mediate adhesion between the cell and virus. Here we report that actin filament nucleation by the viral protein A36 promotes the disengagement of virus attachment. Viruses unable to locally induce actin nucleation displayed significantly reduced release and particles were found trapped in small pits at the plasma membrane. Mutations in luminal viral proteins that disrupt attachment identified an alternative route to virus release, bypassing the requirement for actin nucleation. Our results suggest that untethering virus attachment to the cell surface is a rate-limiting step during exocytic release of vaccinia virus. We have elucidated that the force of actin nucleation is the primary mechanism that operates to relieve these interactions.

## Introduction

Crossing the membrane of host cells, either during entry or escape, is a major obstacle facing prospective viral pathogens during infection. Enveloped viruses are released from cells by either the acquisition or loss of an outer membrane and both strategies pose unique challenges to the final separation of pathogen from host. Where viruses gain a membrane, for example influenza virus and human immunodeficiency virus, a tight association must be formed between assembling viral complexes and the internal surface of the cell membrane that is loaded with viral envelope proteins [1], [2]. As the budding virus emerges into the extracellular space, membrane scission must take place, an energetically difficult event [3]. Other viruses, including herpes simplex virus, acquire a double membrane during morphogenesis that is tightly complexed by viral protein interactions across the luminal space [4]. Upon reaching the cell surface, an exocytotic membrane fusion event is followed by the peeling away of the outer vesicle membrane accompanied by the disengagement of virus–cell associations. Mature enveloped virions are then free to diffuse in the extracellular space. The release of extracellular enveloped virus (EEV), the morphological variant implicated in cell-to-cell transmission of vaccinia virus (VACV), operates through a membrane-loss mechanism. Intracellular enveloped viruses (IEV) arrive at the plasma membrane where the outer of two early endosome or trans-Golgi derived membranes fuses with the plasma membrane forming cell-associated enveloped virus (CEV) [reviewed in 5]. Extracellular CEV remain associated with host cells and this attachment is likely to be mediated by viral envelope proteins [6], [7], [8]. For example, the viral proteins B5, A33 and A34 (encoded by the B5R, A33R and A34R genes, respectively) contain significant luminal regions [9], [10], [11], [12]. In support of a role for these luminal domains in virus–host cell adhesion, a number of mutations in these proteins have been documented that lead to increased EEV release (B5^P189S^, A34^K151E^ and a C-terminal deletion in A33) [6], [13], [14]. While CEV are able to maintain a tight affinity to the surface of infected cells, over time they are untethered and free to access the extracellular space, though how the affinity is regulated remains largely unknown. A number of lines of evidence demonstrate that host-signalling pathways affect the dissociation of VACV. For example, cell lines of different origin display significantly altered kinetics of EEV release that is independent of the total amount of virus made [15]. Inhibition of epidermal growth factor receptor reduces EEV from VACV IHD-J strain [16]. Finally, EEV levels are reduced in the absence of the phosphoinositide 5-phosphatase SHIP2 [17]. These data further support the hypothesis that the generation of enveloped virus and the untethering of enveloped virus are regulated independently. Inhibition of Abl tyrosine kinases also results in reduced EEV [18], [19], although at this stage it is unclear as to whether this is a specific defect in EEV dissociation or a more general defect in virus morphogenesis.

The viral transmembrane protein A36 is currently the only Abl substrate that has been implicated in VACV morphogenesis, although its role in Abl-mediated release is unknown [18], [19], [20]. Of the integral viral proteins localised to IEV, A36 is unusual in that it is expressed exclusively in the outer of the two trans-Golgi-derived membranes [21], [22]. Consequently, it is present beneath CEV once the outer virus membrane fuses and becomes contiguous with the plasma membrane, and accordingly is absent from released EEV. Unlike B5, A33 and A34, the bulk of the A36 protein extends into the cytoplasm. Here it mediates interactions with the microtubule cytoskeleton via WE/WD motifs [23], [24], [25], and the actin cytoskeleton via the phosphorylation of tyrosine residues at positions 112 and 132 (A36^Y112^, A36^Y132^ respectively) [26], [27], [28]. Src- and Abl-family kinases instigate the phosphorylation of A36^Y112^ and A36^Y132^, which generates binding sites for the SH2 domains of Nck and Grb2 adaptor proteins that combine to stabilise N-WASP, a potent activator of the Arp2/3 complex [20], [26], [28], [29], [30]. Activation of the F-actin branching activity of the Arp2/3 complex beneath CEV promotes virus motility that is restricted to the plane of the cell membrane and results in thin virus-tipped membrane protrusions. Although Src- and Abl-family kinases act redundantly to phosphorylate A36 [19], [20], inhibition of Abl-family kinases alone with the specific inhibitor imatinib results in reduced EEV release without affecting actin nucleation [19], [31]. Localised actin nucleation expedites the dispersal of CEV to neighbouring cells and also facilitates super-repulsion, which has been proposed to account for the rapid cell-to-cell transmission of virus that outpaces replication dynamics [32]. Super-repulsion occurs when either CEV or EEV contact the surface of early-infected cells, are repelled by A36-induced actin-based motility and leapfrog to uninfected cells. This process is dependent on early-stage expression of A36 in the recipient cell membrane, before infectious progeny are produced.

Here we show that introducing both Y112F and Y132F mutations into A36 leads to a severe decrease in the production of EEV. In the absence of virus-induced actin nucleation, virus particles remained trapped at the plasma membrane in small invaginations.

When parental VACV was examined using three-dimensional structured illumination microscopy (3D-SIM), it was found that upon reaching the surface, A36 redistributed to a discrete region beneath CEV, reducing contact between extracellular virus and the host cell. This redistribution was dependent on actin-based motility but could also be phenocopied by a mutation in the luminal domain of the envelope protein B5. In our model, following the fusion of IEV at the plasma membrane, CEV remain tethered to the cell in tight membrane pits through interactions between viral proteins. Tethering can be relieved either by mutations in the luminal domains of viral proteins that disrupt these interactions, or by the force supplied by localised actin nucleation.

## Results

### A36 Y112 and Y132 play an essential role in EEV release

**Figure 1:**
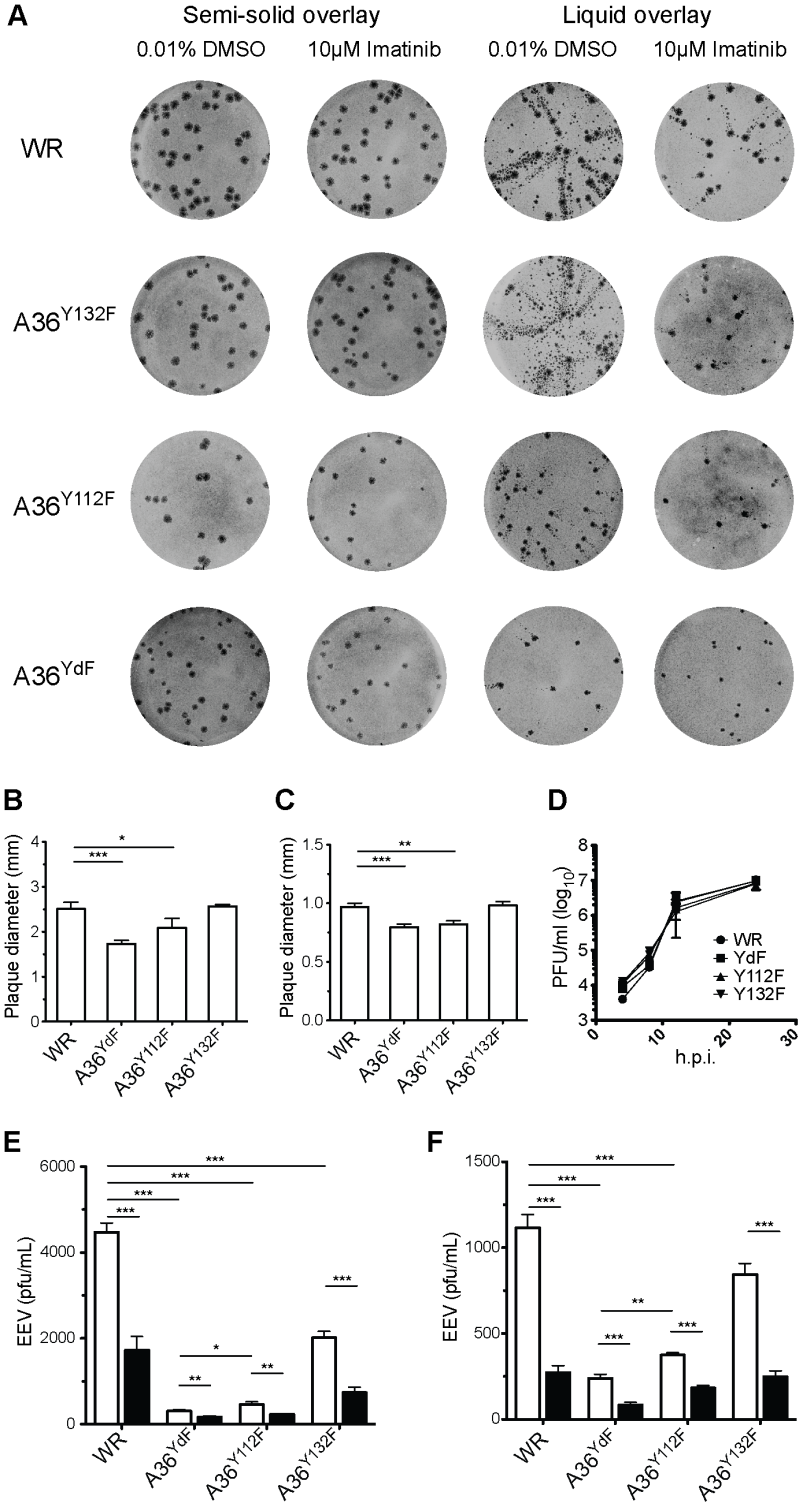
Plaque size and release of VACV is inhibited by mutation of Y112 and Y132 residues in A36. (**A**) BSC-1 cells were infected with the indicated viruses, incubated with a semi-solid (plaque assay, 1.5% CMC) or liquid (comet assay, DMEM) overlay, with either imatinib or carrier (DMSO), stained at 72 hpi. (**B**) Average plaque size of WR, A36^Y112F^, A36^Y132F^ and A36^YdF^ in BSC-1 or (**C**) NIH 3T3 cells. Error bars represent s.e.m. from 50 plaques. (**D**) Single-step growth analysis of VACV strains. Monolayers of BSC-1 cells were infected with the indicated viruses at a MOI of 5. At 4, 8, 12 and 24 hpi cells and supernatants were harvested and virus titres were determined by titration with BSC-1s. The average titres of two independent experiments are plotted at each time point. (**E**) Levels of infectious EEV in supernatants collected 16 hpi from BSC-1 cells or (**F**) NIH3T3 cells infected at an MOI of 0.1 and overlayed with DMEM containing 0.01% DMSO (unfilled) or 10 µM imatinib (filled). Error bars represent s.e.m. from 3 replicate wells in 3 independent experiments. P values of <0.05, <0.01 and <0.001 are represented by *, ** and ***, respectively.

Actin-based motility appears to have evolved independently in a diverse range of pathogenic bacteria and viruses, and it is speculated to contribute to virulence in a context-specific manner [33], [34]. To better understand the role of pathogen-induced actin nucleation in the replication and spread of VACV, we tested the effects of blocking the phosphorylation of A36^Y112^ and A36^Y132^ on the release of EEV. These residues are critical to the canonical pathway whereby VACV induces actin nucleation by recruitment and activation of the Arp2/3 complex. Plaque assays performed under semi-solid overlay are used to quantitate replication dynamics and cell-to-cell transmission. When performed under a liquid overlay, these are termed ‘comet’ assays and the efficiency of EEV release can be qualitatively assessed by the formation of satellite plaques that disperse from primary plaques due to the action of convection currents [6], [35]. Plaque assays were performed in BSC-1 cells with VACV Western Reserve (WR, parental strain), A36^Y112F^, A36^Y132F^ and A36^YdF^ ( = A36^Y112F/Y132F^) to assess cell-to-cell spread (Figure 1A). The A36^YdF^ virus yielded plaques significantly smaller than WR, in broad agreement with previous reports [36]. Mutation of Y112 alone also had a significant effect on plaque size whereas mutation of Y132 alone resulted in plaques not significantly different to those of WR (Figure 1B). Similar results were observed when using NIH3T3 cells (Figure 1C). Reduction in plaque size is likely to be due to the loss of actin-based motility, potentially by its role in facilitating cell-to-cell spread by super-repulsion [32]. A36^YdF^ and A36^Y112F^ viruses do not activate the Nck pathway, which is both necessary and sufficient for actin-based motility, whereas mutation of Y132 leads to a disruption in the dynamics of N-WASP recruitment and a reduction in the efficiency of the initiation of actin-based motility [26], [28].

We next subjected the A36 mutant strains to comet assays to assess EEV release. A reduction in the extent of comet formation was observed in all strains carrying Y/F substitutions (Figure 1A). The greatest reduction was observed in cells infected with A36^YdF^, followed by A36^Y112F^, and only a minor reduction was seen with A36^Y132F^. To support these findings and quantify the effects of A36 phosphorylation on virus release, EEV release assays were performed in BSC-1 cells. We observed the same trend in EEV release as in the comet assays, with the greatest inhibition of release (approximately 10-fold) seen with A36^YdF^ and a significant inhibition seen when Y112 was mutated (Figure 1E). These findings were replicated when release assays were performed in NIH3T3 cells (Figure 1F). Disruption of A36 does not result in early defects in wrapping and morphogenesis and the Y112 and Y132 residues are not required for microtubule-dependent delivery of IEV to the cell surface [21], [23], [24], [37], [38], [39]. Single-step growth curves did not reveal significant differences in the replication dynamics between WR, A36^Y112F^, A36^Y132F^ and A36^YdF^ (Figure 1D). Thus, any differences observed in EEV release for these viruses cannot be accounted for by earlier morphogenesis or transport defects.

Abl kinases regulate at least two distinct steps during VACV replication: EEV release and actin-based motility of CEV, in the latter case through A36 phosphorylation [19], [20]. The effects of abrogating release by mutation of Y112 or Y132 are consistent with A36 fulfilling the role as the Abl-dependent mediator of virus release. To test this role, we examined the effects of imatinib in our various mutant backgrounds. Imatinib is a kinase inhibitor that blocks the ATP binding site on Abl-family kinases and has remarkable specificity displaying no detectable activity against Src-family kinases [20], [31]. Surprisingly, we found that regardless of the integrity of A36 residues Y112 and Y132, treatment with imatinib led to a significant reduction in comet formation and an approximately 2- to 3-fold decrease in EEV (Figure 1A, 1E, 1F). These results demonstrate a role for A36 tyrosine phosphorylation in release that is independent of the effects of imatinib. Although we cannot exclude that Abl kinases regulate virus release via A36 phosphorylation, if they do so they act redundantly with Src-family kinases, as they do in the initiation of actin-based motility.

### A second site mutation in B5 restores EEV release independently of A36 Tyr substitutions

**Figure 2:**
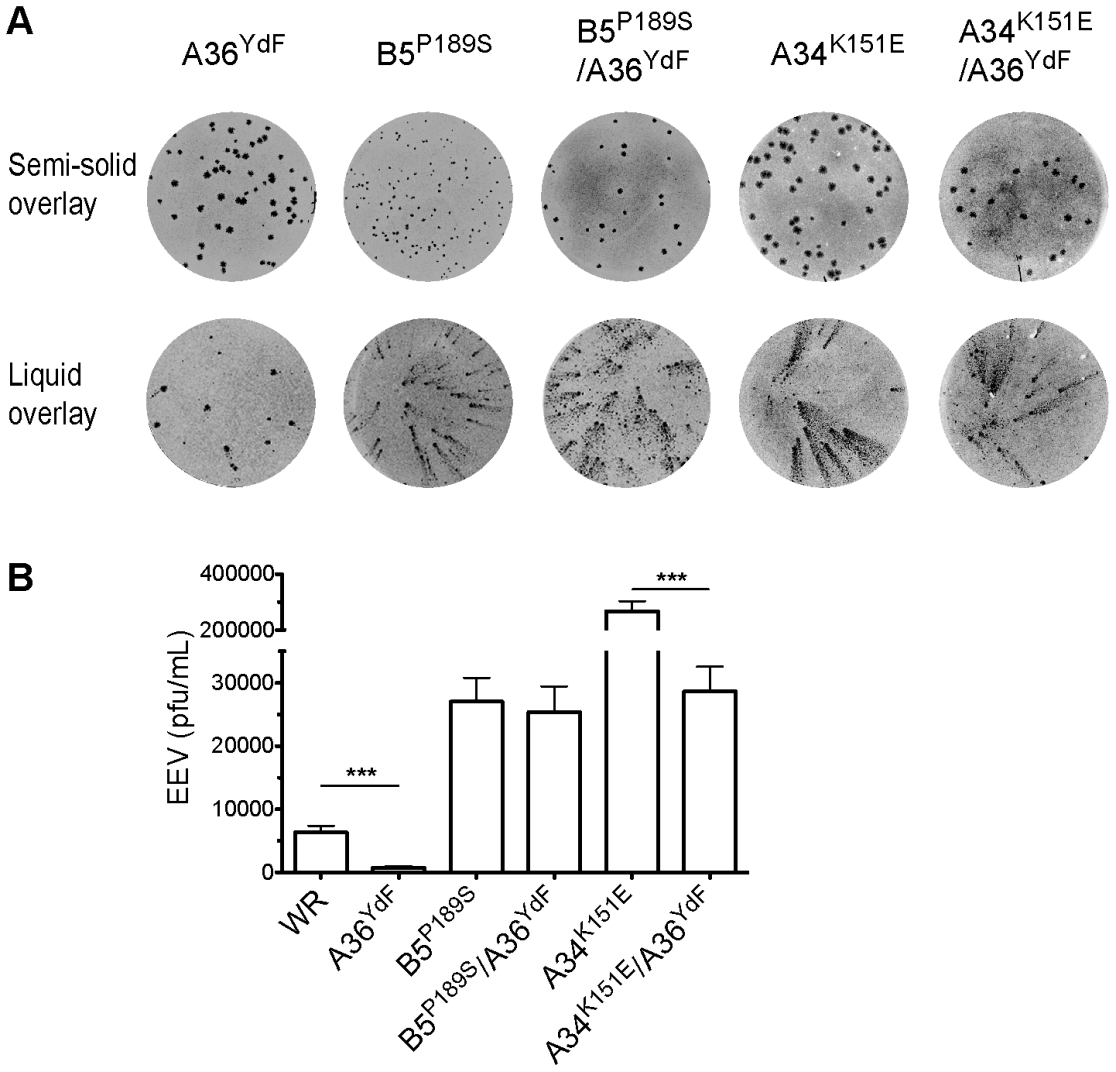
A second site mutation in B5 restores EEV release in A36^YdF^. (**A**) Plaque (semi-solid overlay) and comet (liquid overlay) assays of A36^YdF^, B5^P189S^, B5^P189S^/A36^YdF^, A34^K151E^ or A34^K151E^/A36^YdF^ stained at 72 hpi. (**B**) Levels of infectious EEV in supernatants collected at 16 hpi from BSC-1 cells infected at an MOI of 0.1. Error bars represent s.e.m. from 3 replicate wells in 3 independent experiments. P values of <0.05 and <0.01 are represented by * and **, respectively.

Deletion of genes encoding envelope-specific proteins can increase (A33R, A34R) or decrease (B5R, F13L) production of EEV, often due to earlier defects in morphogenesis [40], [41], [42], [43]. While our results are the first to implicate A36 tyrosine phosphorylation in virus release, it has previously been shown that deletion of A36 results in a dramatic reduction in EEV [37]. This is unsurprising given the essential role this protein plays in delivering IEV to the cell surface through kinesin-1 based transport; however, Y112 and Y132 play no part in this process [23], [24], [25]. Inhibition of virus release in an A36 deletion strain can be derepressed, and significantly enhanced, by second-site mutations in genes that encode the envelope-specific proteins B5 (P189S substitution) and A33 (C-terminal truncations) [14]. A point mutation in A34R (K151E), identified in the IHD-J strain, also significantly enhances EEV release in a WR background [6], [19]. We tested whether the B5^P189S^ or A34^K151E^ alleles would also promote EEV release in an A36^YdF^ background. Both B5^P189S^ and A34^K151E^ led to substantial increases in comet formation both in a parental and A36^YdF^ background (Figure 2A). EEV release assays revealed that the B5^P189S^ virus generated 5-fold more infectious EEV than the parental WR strain and 40-fold more than A36^YdF^ (Figure 2B). There was no significant difference in EEV release between B5^P189S^ in a WR background or an A36^YdF^ background. The A34^K151E^ virus produced 50-fold more infectious EEV than WR and 400-fold more than A36^YdF^ (Figure 2B). In contrast to the B5^P189S^ virus, a significant reduction (10-fold) in EEV was observed in A34^K151E^/A36^YdF^ virus compared to A34^K151E^ (Figure 2B). Therefore, in a background where actin-based motility is intact (WR and A34^K151E^) [17], [44], the A36^YdF^ mutation potently supresses EEV release. Conversely, in a B5^P189S^ background, which is strongly deficient in actin-based motility owing to a failure of kinase activation, the A36^YdF^ allele has no effect on EEV release [30].

### Inhibition of virus-induced actin nucleation results in defects in EEV release

**Figure 3:**
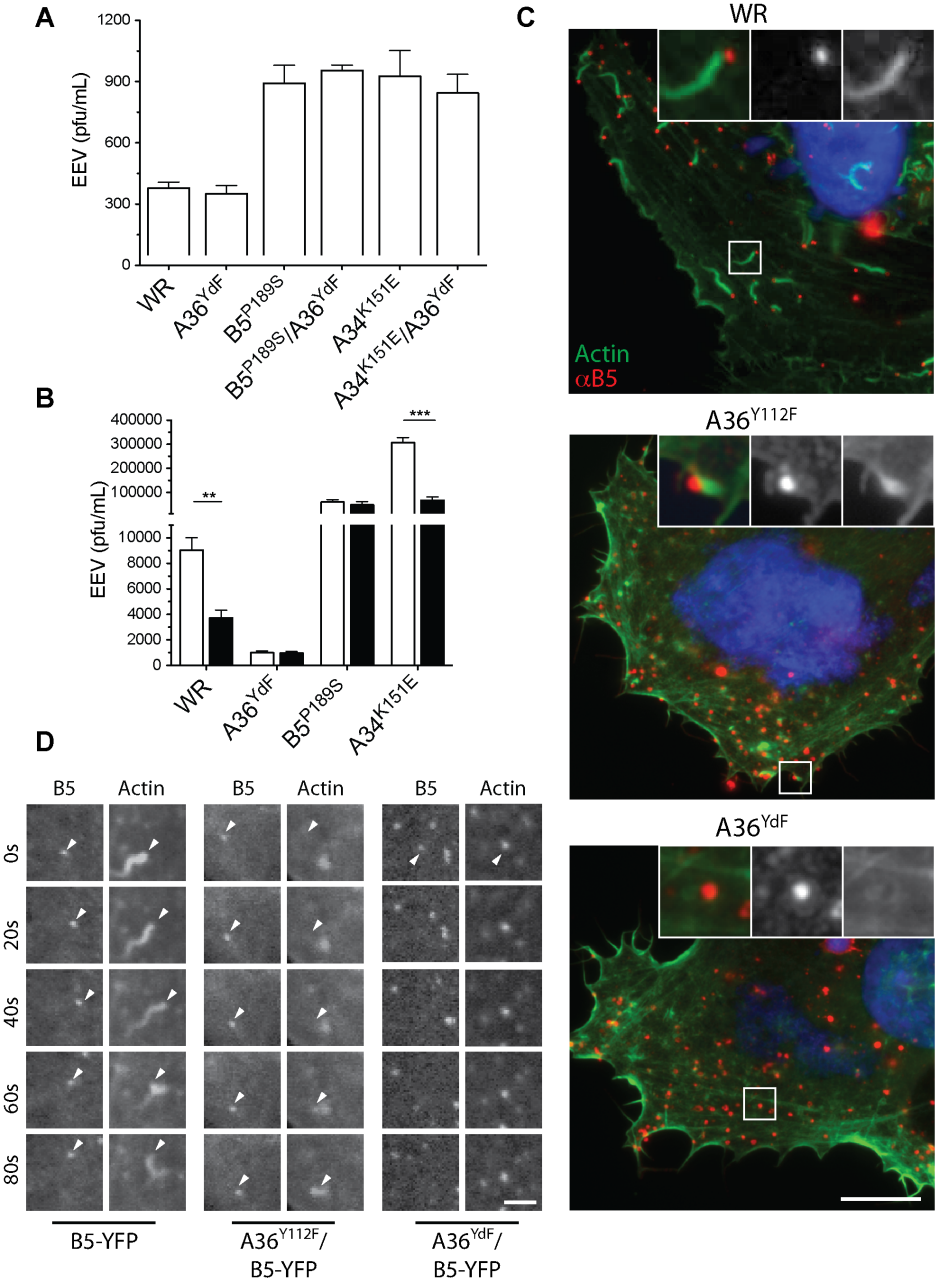
Actin polymerisation is required for efficient EEV release. Infectious EEV levels in supernatant collected at 16 hpi from (**A**) Nck-null cells infected with WR, A36^YdF^ B5^P189S^, B5^P189S^/A36^YdF^, A34^K151E^ or A34^K151E^/A36^YdF^ at an MOI of 0.1; (**B**) BSC-1 cells infected with WR, A36^YdF^, B5^P189S^ or A34^K151E^ at an MOI of 0.1 and overlayed with DMEM containing 0.01% DMSO (unfilled) or 0.1 µg/ml Cyt D (filled). Error bars represent s.e.m. from 3 replicate wells in 3 independent experiments. P values of <0.01 and <0.001 are represented by ** and ***, respectively. (**C**) Fluorescent micrographs of HeLa cells infected with WR, A36^Y112F^ and A36^YdF^. Robust F-actin comets are associated with WR and localised accumulation of actin is observed sporadically with A36^Y112F^ (insets). Extracellular virus is detected with B5 antibody (red, non-permeabilised cells) and actin with phalloidin (green). Scale bar = 10 µm. (**D**) Confocal micrographs of HeLa cells infected with B5-YFP, A36^Y112F^/B5-YFP and A36^YdF^/B5-YFP and expressing Lifeact-mRFP. Distinct F-actin comets are associated with both B5-YFP and A36^Y112F^/B5-YFP, while colocalisation between F-actin and A36^YdF^/B5-YFP occurs stochastically as viruses move over previously existent F-actin structures. Scale bar = 3 µm.

Our results indicate that phosphorylation of A36 is required for efficient EEV release but do not discriminate whether this is due to the induction of localised actin polymerisation or signalling via A36 through another mechanism. We therefore tested the effects of inhibiting actin nucleation on EEV production using two different approaches. Nck-null mouse embryonic fibroblasts do not support virus-associated actin nucleation due to the essential requirement of this adaptor protein in the recruitment of N-WASP and subsequent activation of the Arp2/3 complex [26], [28], [45]. Nck-null cells infected with WR or A36^YdF^ released similar levels of EEV (Figure 3A). Nck-null cells infected with B5^P189S^, B5^P189S^/A36^YdF^, A34^K151E^ and A34^K151E^/A36^YdF^exhibited similar levels of EEV release, approximately 3-fold greater than WR and A36^YdF^. We further tested the role of virus-induced actin nucleation by treating infected cells with cytochalasin D (Cyt D), a potent inhibitor of actin polymerisation that caps the fast growing ends of actin filaments. Previous studies have shown that Cyt D significantly reduces WR EEV while having little effect on morphogenesis or formation of CEV [15], [39], [46]. Treatment with Cyt D resulted in a 3-fold reduction in EEV release from cells infected with WR or A34^K151E^ (Figure 3B). In contrast, Cyt D had little impact on EEV production in cells infected with A36^YdF^ or B5^P189S^. Collectively, these data suggest that in the absence of virus-induced actin nucleation, the release of EEV is independent of the status of A36 Y112 and Y132 residues. Hence, the deficiency in EEV release observed for A36^YdF^ can be replicated by Cyt D treatment, and in a cell line where actin-based motility is blocked, inhibition of EEV release due to the YdF mutation is not observed.

### A36^Y112F^ induces localised F-actin accumulation

Our results show a correlation between actin-based motility and release of EEV, with one anomaly. The A36^Y112F^ virus displays a small, but significant, increase in virus release compared to A36^YdF^ (Figure 1E, 1F), yet both strains have been characterised as deficient in actin-based motility [26]. To resolve this paradox, we re-examined A36^Y112F^-infected cells and compared these with WR- and A36^YdF^-infected cells. Consistent with previous reports we were unable to detect virus-associated F-actin tails of 3–4 µm in length, which are typical of WR infection, in A36^Y112F^-infected cells (Figure 3C) [28]. However, we did observe F-actin accumulations that colocalised with extracellular A36^Y112F^ virus, albeit infrequently. Small clumps of F-actin accumulation are observed in VACV-infected and uninfected cells at a low frequency, so to determine whether the observed colocalisations were stochastic events or represented viral-induced actin nucleation, we imaged cells with live microscopy (Figure 3D and Video S1, S2, S3). In cells infected with A36^YdF^ (n = 9), no motile virus particles were associated with F-actin. In cells infected with A36^Y112F^ (n = 9), six cells had at least one example of virus motility associated with transient F-actin accumulation over the course of two minutes. This was in contrast to actin tails that were associated with parental WR virus, which were robust and longer lived (Figure 3D). Taken together, these results confirm a correspondence between the ability to stimulate actin nucleation, albeit weakly in the case of A36^Y112F^, and defects in EEV release.

### A36^YdF^ enveloped virus localises to pits at the plasma membrane

**Figure 4:**
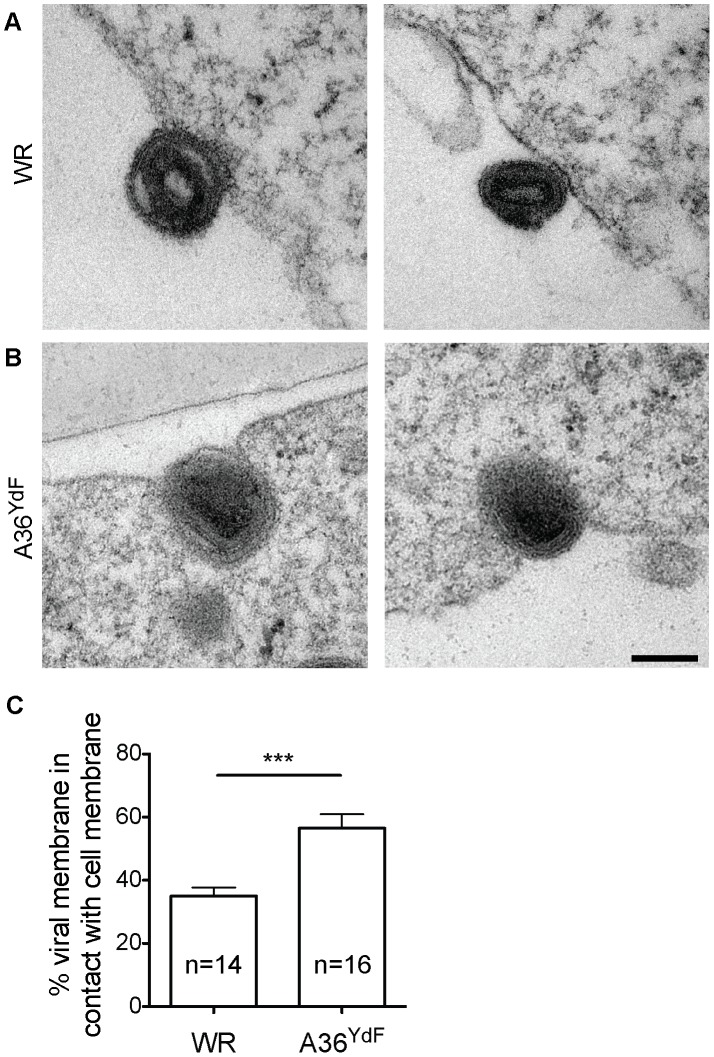
A36^YdF^ is invaginated at the cell membrane during egress. Representative transmission electron micrographs of BSC-1 cells infected with (**A**) WR and (**B**) A36^YdF^ at an MOI of 5 and fixed at 9 hpi. In contrast to WR, distinct membrane pits containing virus are apparent during A36^YdF^ virus egress. Scale bar = 0.2 µm. (**C**) Average percentage of viral membrane circumference in contact with cell membrane calculated from 14 (WR) or 16 (YdF) viruses randomly selected from the transmission electron micrographs. *** = P value of <0.001.

In order to disclose the mechanism by which EEV release is disrupted in A36^YdF^, we examined infected cells by transmission electron microscopy (TEM). The majority of exiting enveloped viruses were found loosely associated with the plasma membrane in WR infected cells (Figure 4A), whereas A36^YdF^ viruses were predominantly contained in membrane pits at the surface of infected cells (Figure 4B). A36^YdF^ viruses that remained at the cell surface were identified as CEV as the outer viral membrane was observed to be contiguous with the plasma membrane [47], [48]. The proportion of the CEV envelope in contact with the cell membrane was measured and was found to be significantly different between WR and A36^YdF^. A36^YdF^ CEV displayed greater contact on average with the host membrane than the parental strain (Figure 4C). CEV residing in plasma membrane pits have been previously documented when cells infected with IHD-J, a strain harbouring the A34^K151E^ substitution, were treated with Cyt D (see Figure 4A in [15]). These results suggested that virus-induced actin nucleation might function to expel CEV from membrane pits leading to release of EEV.

### A36 redistribution upon virus exit is inhibited in the absence of actin nucleation

**Figure 5:**
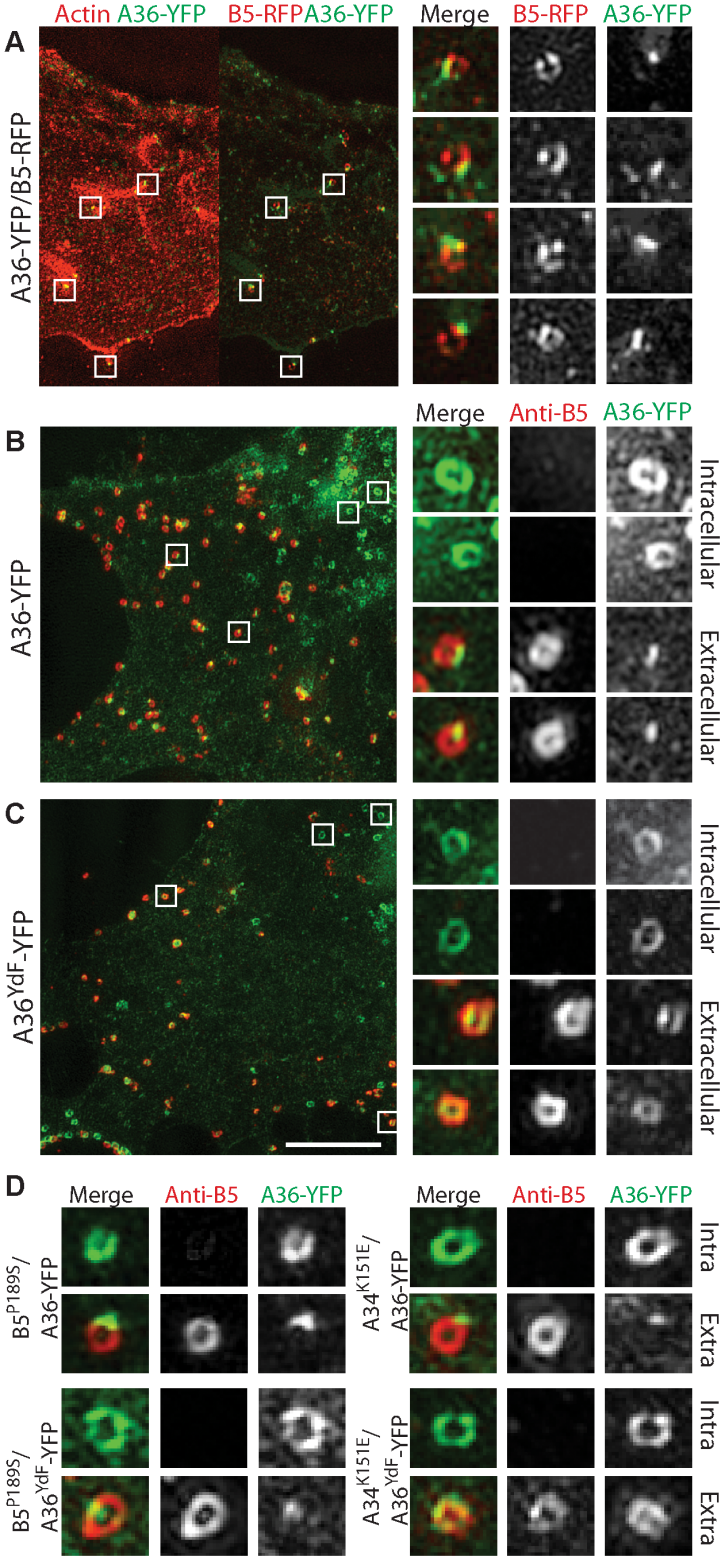
Redistribution of A36 during virus exit. (**A**) 3D-SIM fluorescent micrograph of A36-YFP/B5-RFP infected BSC-1 cell. Virus particles associated with F-actin (red, left panel; visualised with Lifeact-cerulean) show polarisation of A36 (green) whereas B5 (red, right panel) localises to the CEV circumference (close-ups). 3D-SIM fluorescent micrographs of (**B**) A36-YFP, (**C**) A36^YdF^-YFP in non-permeabilised BSC-1 cells probed with rat anti-B5 primary antibody and Alexafluor 568 anti-rat secondary antibody. Close-ups show A36 (green) localisation in two representative intracellular (no B5 visible) and extracellular virus particles. (**D**) 3D-SIM fluorescent micrographs of representative intracellular and extracellular B5^P189S^/A36-YFP, B5^P189S^/A36^YdF^-YFP, A34^K151E^/A36-YFP or A34^K151E^/A36^YdF^-YFP viruses in non-permeabilised BSC-1 cells probed with rat anti-B5 primary antibody and Alexafluor 568 anti-rat secondary antibody. All close-ups from larger panels are arranged in order from top to bottom. Scale bar = 5 µm.

**Figure 6:**
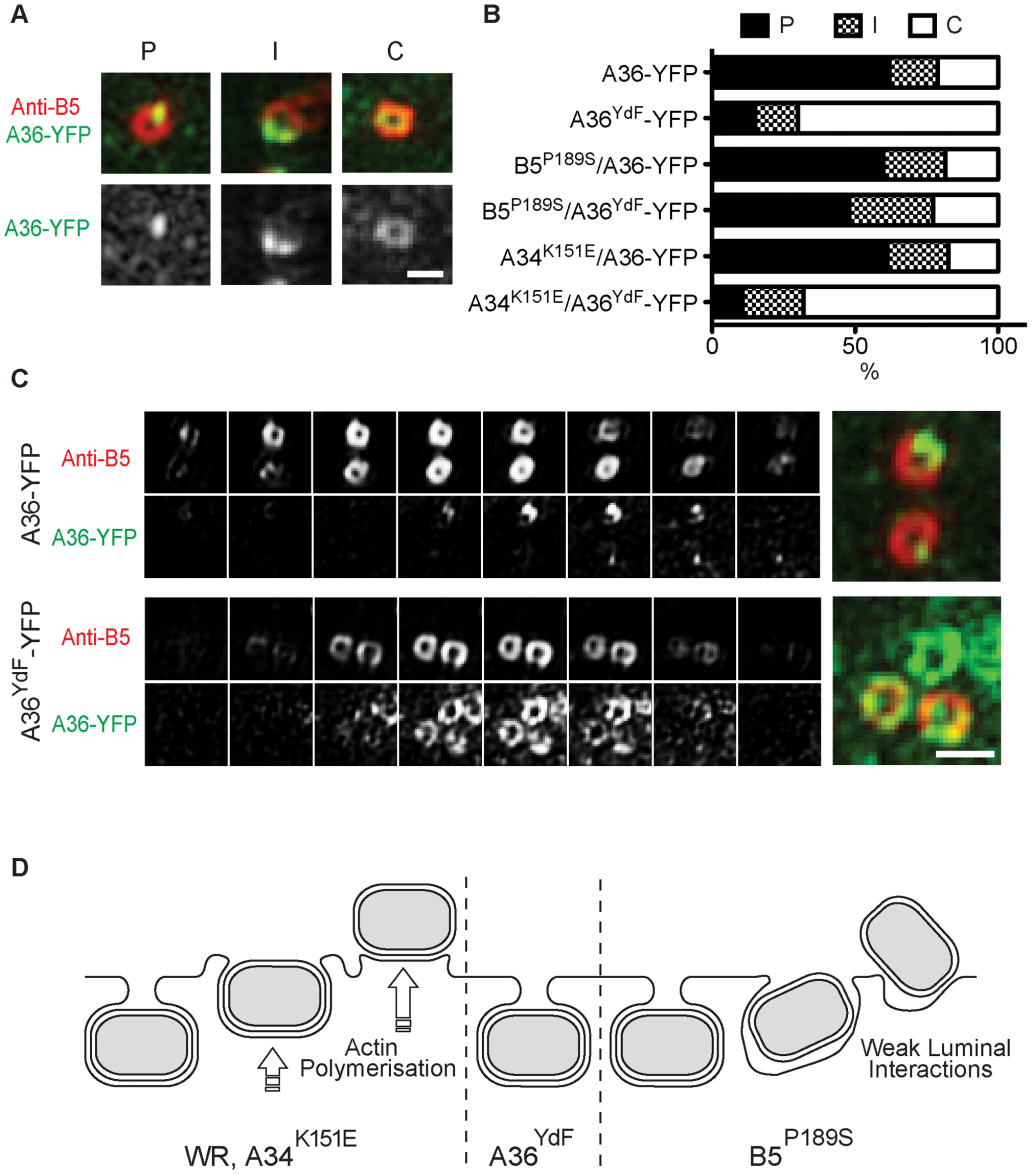
Quantification of polarisation of A36. (**A**) Examples of the three categories for A36 distribution in 3D-SIM images: P = Polarised; I = Intermediate; C = Circular, and (**B**) percentage of viruses in each category for cells infected with A36-YFP, A36^YdF^-YFP, B5^P189S^/A36-YFP, B5^P189S^/A36^YdF^-YFP, A34^K151E^/A36-YFP or A34^K151E^/A36^YdF^-YFP. (**C**) Z-slices of representative A36 distributions in single A36-YFP and A36^YdF^-YFP virus particles. Scale bars = 0.5 µm. (**D**) Schematic of proposed events at the cell membrane for WR, A36^YdF^, B5^P189S^ or A34^K151E^ during egress.

Despite the high resolution afforded by TEM, the extremely small portion of the total cell volume that is included in an ultrathin section does not provide sufficient data for extensive quantitative comparisons between strains. Fluorescence microscopy of fluorescently-tagged or immunolabelled proteins is a more efficient way to visualise protein localisation and this approach is also amenable to labelling of non-permeabilised cells to distinguish extracellular epitopes. Standard wide-field or confocal imaging is, however, limited by the diffraction of light that poses a limit on resolving power to approximately 250 nm. To exploit the advantages of fluorescence microscopy and simultaneously resolve virus particle morphology we utilised 3D-SIM super-resolution microscopy [49]. We generated recombinant viruses that inserted A36-YFP or A36^YdF^-YFP into the endogenous locus; these were subsequently combined with B5^P189S^, A34^K151E^ and B5-mRFP (monomeric red fluorescent protein) alleles or transgenes. Initially, cells expressing Lifeact fused to cerulean fluorescent protein (to visualise the actin cytoskeleton) were infected with A36-YFP/B5-mRFP virus. Lifeact is a 17 amino acid peptide that binds to filamentous actin [50]. Using 3D-SIM, envelope proteins A36 and B5 resolved as hollow spherical structures that corresponded to IEV [data not shown and 51]. When A36 was localised to virus-tipped F-actin tails, a redistribution was apparent as A36 concentrated to discrete regions adjacent to the virus particles, whereas B5 remained distributed along the circumference of virus particles (Figure 5A). This is consistent with previous electron microscopy studies which have demonstrated that unlike B5, A36 is excluded from the inner of the two trans-Golgi derived viral membranes [22]. To further support our electron microscopy data, we compared the localisation of A36-YFP with A36^YdF^-YFP, while distinguishing between extracellular and intracellular enveloped virus with an antibody label (Figure 5B, 5C, 6C). We were able to observe a trend of A36-YFP polar redistribution upon fusion with the plasma membrane that was inhibited in the A36^YdF^ background. Thus, in the absence of A36 tyrosine phosphorylation and actin nucleation, close contact is maintained between extracellular virus (labelled with anti-B5) and the surface of the cell (labelled with A36-YFP). Analysis of individual z-planes in a 3D reconstruction revealed contact between the virus and cell was limited to a few planes in A36-YFP, but in A36^YdF^-YFP A36 colocalised with exposed B5 over the majority of a CEV particle (Figure 6C), reflecting the TEM phenotype. A36 was frequently polarised at the surface of both B5^P189S^/A36-YFP and B5^P189S^/A36^YdF^-YFP enveloped virus (Figure 5D). Thus introduction of the B5^P189S^ allele restored A36 polarisation in a YdF background. In contrast, the polarisation of A36 in A34^K151E^/A36-YFP was inhibited by the YdF mutation (Figure 5D). These data were quantified by enumerating single viruses that corresponded to categories of close, intermediate and loose contact (polar, intermediate and circular A36 distributions). This analysis revealed the majority of A36-YFP, B5^P189S^/A36-YFP, B5^P189S^/A36^YdF^-YFP and A34^K151E^/A36-YFP CEV displayed polar A36 distribution and the majority of A36^YdF^-YFP and A34^K151E^/A36^YdF^-YFP CEV displayed a circular A36 distribution, colocalising with extracellular B5 (Figure 6A, 6B). Collectively these data show that in backgrounds that support actin-based motility, introduction of the YdF mutation leads to viruses getting trapped at the cell membrane with a failure to readily disengage virus–host contact.

## Discussion

### The release of enveloped virus during VACV replication

Substantial evidence supports the critical role of the enveloped form of orthopox viruses in cell-to-cell transmission [52], [53], despite EEV being stoichiometrically the minor infectious form produced during virus replication [54], [55]. Epitopes derived from envelope proteins are major antigens recognised by host immune defences and neutralising antibodies to these antigens are effective in providing protective immunity [56], [57]. Mutations in viral genes that encode envelope-specific proteins also lead to attenuation both *in vitro*, such as reduced cell-to-cell spread measured through plaque assays, and when used to infect animal models [8], [14]. These findings can be explained by effects on transmission by CEV or the speed at which EEV are released from infected cells. While there is evidence that both IMV and IEV are transported to the cell periphery on microtubules, enveloped virus, using A36-mediated transport via kinesin-1, is efficiently translocated to the cell periphery at approximately eight hours post infection. This is the earliest time point that infectious progeny are released [39], [58] and at this time relatively few IMV are dispersed throughout the cell. Additionally, IMV lack a specific mechanism for release from infected cells other than through cell lysis, which occurs only late during infection [56]. Little is known regarding the fusion event between the outer IEV envelope and the plasma membrane leading to the formation of CEV, but this event correlates with a number of changes at the molecular level. These changes include the rapid dissociation of the viral proteins F12 and E2 and the kinesin-1 motor complex [22], [30], [59], [60], followed by recruitment of Src- and Abl-family kinases and the phosphorylation of A36 [19], [20], [27], [30]. Although the exact mechanism that leads to the recruitment of cellular kinases to CEV is unknown, it is dependent on an intact SCR4 domain of B5 which becomes exposed on the cell surface upon viral fusion [30]. During super-repulsion, the B5 SCR4 must also be present on the surface of EEV to efficiently induce actin-based motility on A33/A36 expressing cells [44]. This provides further support for the ability of this domain to induce signalling events across the extracellular space between enveloped virus and the plasma membrane. We have now shown that in the absence of A36 phosphorylation and subsequent localised actin nucleation, CEV remain trapped in pits at the plasma membrane thereby blocking the liberation of EEV from infected cells (Figure 6D). Our model is supported by the strong inhibition in EEV release observed in the A36^YdF^ strain, which can be replicated through blocking actin nucleation by removing Nck or with drug intervention.

Humphries *et al.* [61] have recently described a role for clathrin and the clathrin adaptor protein AP-2 in the regulation of VACV-induced actin nucleation. In the absence of AP-2, actin-based motility is delayed and N-WASP turnover during motility is reduced. Concurrent with these effects was a reduction in A36 polarisation, which strongly supports our findings: disrupting actin nucleation correlates with inhibition of the expulsion of enveloped virus at the cell surface. Even slight differences in the potential of actin nucleation, such as between the A36^Y112F^ and A36^YdF^ strains, results in changes to the efficiency of EEV release. Humphries *et al*. also describe the localisation of N-Wasp, which, like A36, is less polarised when actin nucleation is disrupted, although the temporal dynamics in relation to virus expulsion are not clear, as they were unable to distinguish between extracellular and intracellular virus. With great prescience, Payne [15], upon observing electron micrographs of Cyt D-treated IHD-J infected cells, speculated that “the final separation of EEV from the plasma membrane is probably dependent on functioning microfilaments [actin]” but, as actin-based motility was unknown at the time, concluded that the membrane dynamics associated with cell motility was the ultimate cause.

Inhibiting the action of Abl-family kinases with the specific inhibitor imatinib leads to a reduction in EEV release, implicating these kinases in the regulation of this process. Despite our previous work demonstrating that A36 was a direct Abl substrate [20], we believe the mechanism by which imatinib disrupts virus release is independent of the role of A36 based on the following evidence. Firstly, disruption of Y112 and Y132 (A36^YdF^) leads to a far stronger reduction in EEV (10-fold) than treatment with imatinib (3-fold). Secondly, imatinib inhibits EEV release regardless of the integrity of A36 Y112 and Y132 residues. Therefore, while Abl family kinases may regulate EEV release through phosphorylation of A36, they do so redundantly with Src-family kinases, as we have previously proposed for actin-based motility [20]. The effects of imatinib on virus release suggests another, Src-independent, role for Abl kinases, the mechanism of which remains unknown.

### Tethering function of envelope proteins

To better characterise the mechanism by which actin nucleation promotes virus release, we examined the effects of recombining the A36^YdF^ mutation into high EEV release backgrounds. These included strains carrying mutations in the envelope proteins B5 (B5^P189S^) and A34 (A34^K151E^). The B5^P189S^ virus exhibits high levels of EEV release irrespective of the status of A36 Y112 and Y132 residues [6], [13], [14]. In contrast, the high level of A34^K151E^ EEV release was potently suppressed by the A36^YdF^ mutation. These data show that in strains that activate actin-based motility (WR and A34^K151E^) the YdF mutation suppresses EEV release, but has no effect in cases where actin-based motility is absent (B5^P189S^-infected or Nck null cells) [13], [14], [30]. Although both A34^K151E^ and B5^P189S^ result in high levels of EEV, only the latter bypasses the requirement for actin-based motility to facilitate release. A34^K151E^ appears to exhibit a faster rate of enveloped virus production, but EEV release is nonetheless regulated in the same manner as the parental WR strain [62]. In B5^P189S^ a high rate of EEV release is achieved at a cost to morphogenesis, hence the small plaque phenotype. Collectively, these findings indicate that two pathways can liberate EEV from the surface of cells: localised actin filament nucleation or disruption of viral protein-to-host membrane interactions. The exact nature of these interactions is unclear as few experiments have been performed that are able to distinguish between interactions occurring in *cis* and *trans* in the viral envelope [63], [64], and the pleiotropic roles of these proteins confounds the interpretation of mutant phenotypes. The best evidence for an interaction between the outer viral membranes being mediated by the luminal domains of viral proteins derives from Perdiguero *et al.* [65], who show that the SCR regions of B5 can interact with the ectodomain of A34 in the absence of transmembrane domains. Recent findings on the mechanism of super-repulsion also implicate A33 as a B5-interaction partner. Doceul *et al* [32] demonstrated that expression of only A33 and A36 in the recipient host cell membrane is required for super-repulsion. It was subsequently reported that the B5^P189S^ mutation on the surface of EEV also disrupts super-repulsion actin-based motility [44]. Recently, an interaction was identified between the isolated luminal domain of A33 and a luminal coiled-coiled domain of B5 that lies adjacent to SCR4 [63]. As this interaction is dependent on the coiled-coiled domain being anchored to a membrane, its role in adhesion across the viral envelope membranes is unclear and may represent an independent role for A33 in incorporation of B5 into the envelope, or vice versa [63], [64], [65]. Intriguingly, mutations that increase B5–A33 adhesion result in reduced binding of EEV to host cells suggesting a *cis* interaction in the membrane between these proteins and raising the possibility that A33 might mask and/or regulate the availability of the adjacent SCR4 [66]. Further characterisation of the nature of these interactions may clarify how adhesion between the outer viral membranes is achieved and regulated. In most cases, a trade-off exists between adhesion and efficient wrapping as a subset of high release mutations in envelope proteins result in small plaque phenotypes and these viruses are attenuated when used to infect mice (B5, A33 [8], [13]). Disrupting these interactions via the force of localised actin nucleation offers an elegant solution to unshackling the luminal interactions that, while required for efficient wrapping, need to be released for EEV to be untethered.

What is the role of EEV release during pathogenesis? The literature does not offer a clear consensus as to whether strains that release more EEV are more or less virulent. Mutations isolated in a WR background such as B5^P189S^ and A33 C-terminal truncations lead to increased EEV release but also a reduction in plaque size [13]. Unsurprisingly, viruses carrying these mutations are attenuated when used to infect mice [13]. Alternatively, one can compare orthopox isolates with characteristic EEV release profiles and correlate virulence. A comparison of variola virus strains identified a high correlation between decreased virulence and high EEV release [67]. It has been speculated that the decreased virulence of the high release orthopox strains may facilitate transmission between hosts to the detriment of cell-to-cell transmission within a host [67]. Mice infected with the high-release IHD-J strain, which carries the A34^K151^ allele, display reduced mortality compared to those infected with WR (70% verses 85% lethality over 3 weeks post-infection) [52], [54]. However, WR, which has low EEV release, may be the exception rather than the rule; a broader comparison of VACV strains show a positive correlation between EEV release and the ability of the virus to disseminate within a host and cause lethality [54]. All of these studies are limited by the use of animals that have not been verified as a natural host and may therefore not preserve aspects of endemic host–pathogen interactions during *in vivo* spread and transmission.

### Viral-induced F-actin nucleation

The first virus characterised as capable of undergoing actin-based motility was VACV, but its role in enhancing infection outcomes has only recently become clear. Virus plaques formed by A36^YdF^ are reduced in size, attesting to the role of these residues in enhancing cell-to-cell spread ([36], this study) and enabling super-repulsion [32]. Here we show that inhibition of A36-induced actin nucleation results in a severe reduction in the release of infectious EEV from infected cells, measured by liquid plaque assay or directly quantified. It should not be surprising that once poxviruses evolved a mechanism to exploit host actin dynamics, this mechanism might be co-opted for other functions. For example, localised actin nucleation may have evolved to untether EEV and then subsequently have been exploited to mediate super-repulsion, or vice versa. In principle, the direction of the co-opting of function could be discriminated by the examination of the promoter activity of A36R homologues; if actin nucleation evolved to facilitate EEV release then distantly related homologues might be expected to lack early stage transcriptional activity. Although A36 is not highly conserved at the sequence level within the *Chordopoxvirinae* subfamily, it is at the functional level. For example, a diversity of poxviruses encode proteins at a homologous locus that are able to restore actin-based motility in a VACV ΔA36 strain despite exhibiting as little as 9% homology at the amino acid level [68]. Owing to the broad definition of early and late promoter consensus motifs [69], [70], a preliminary inspection to identify the type of promoters in the A36R homologues was unsuccessful. Functional studies of the activities of these promoters would potentially resolve this issue. Future research may identify yet further functions for VACV-induced actin nucleation in facilitating virus spread. Pathogen-induced actin nucleation has evolved independently in a number of viral and bacterial lineages, but the role of actin nucleation in pathogenesis varies substantially [71], [72]. During the colonisation of the intestinal tract by Enteropathogenic *Escherichia coli*, extracellular bacteria are thought to attach to the epithelium by the formation of F-actin-rich pedestals, utilising a cascade bearing remarkable similarity to VACV-induced actin nucleation [73]. *Listeria monocytogenes* stimulates polarised actin nucleation at the surface of intracellular bacteria promoting motility within the cell and forming long membrane extensions that may enhance infection of neighbouring cells [74]. Similar structures are observed in VACV infected cells, albeit tipped by an extracellular pathogen, and these are likely to also facilitate cell-to-cell spread during VACV infection. Herpesviruses must also resolve the untethering luminal interactions during de-envelopment from the nuclear membrane and exit at the plasma membrane. Although very little is know regarding these processes there are remarkable similarities to VACV escape [4]. Whether herpesvirus and VACV have evolved similar mechanisms to release virus particles must await further studies, currently F-actin has not been localised to herpesvirus particles during release [75].

A close parallel to the role of actin nucleation that we describe here for EEV release may be clathrin-mediated endocytosis. Here actin filament nucleation at the neck of endocytic vesicles exhibits sufficient force to promote internalisation and membrane scission [76]. This pathway is used during influenza virus entry, which is also sensitive to Cyt D [77]. Vaccinia virus EEV release operates in reverse, the nucleator is localised to the intracellular cargo and the force of actin nucleation peels away the outer envelope, expelling the virus particle to the surface of the cell. Thus a similar intermediate, a recently internalised influenza virus particle or a recently egressed VACV CEV, will resolve in opposite directions depending on where the force is applied.

## Materials and Methods

### Cells

African green monkey kidney cells (BSC-1), murine embryonic fibroblasts (NIH3T3) and Nck-null NIH3T3 cells were maintained in Dulbecco's Modified Eagle's Medium (DMEM; Invitrogen) supplemented with 5% foetal bovine serum (FBS), 292 µg/ml L-glutamine, 100 units/ml penicillin and 100 µg/ml streptomycin (DMEM-FPSG) at 37°C and 5% CO_2_.

### Viruses

Vaccinia virus strain Western Reserve (WR) and the mutant viruses A36^YdF^, A36^Y112F^, A36^Y132F^ and B5-YFP have been described previously [26], [59]. A36-YFP, A36^YdF^-YFP, A36-YFP/B5-mRFP, B5^P189S^, B5^P189S^/A36^YdF^, A34^K151E^ and A34^K151E^/A36^YdF^, A36^YdF^/B5-YFP and A36^Y112F^/B5-YFP were prepared as described previously [28], [59], [78], [79]. Briefly, plasmids containing recombination cassettes with either the point mutations or fluorescent protein sequences flanked by left and right arm regions homologous to the desired area of insertion were prepared. BSC-1 cells were infected with VACV WR and transfected with the relevant vector using Lipofectamine (Invitrogen), as described by the manufacturer, for production of A36-YFP, A36^YdF^-YFP, B5^P189S^ and A34^K151E^, or infected with A36^YdF^ or A36^Y112F^ and transfected with the relevant vector for production of B5^P189S^/A36^YdF^, A34^K151E^/A36^YdF^, A36^YdF^/B5-YFP and A36^Y112F^/B5-YFP. For the other fluorescent viruses, BSC-1 cells were infected with A36-YFP and transfected with the relevant vector for production of A36-YFP/B5-mRFP, B5^P189S^/A36-YFP and A34^K151E^/A36-YFP, or infected with B5^P189S^ or A34^K151E^ and transfected with the A36^YdF^-YFP vector to produce B5^P189S^/A36^YdF^-YFP and A34^K151E^/A36^YdF^-YFP, respectively. At 24 hours post infection (hpi) cells were scraped and recombinant viruses purified by three rounds of plaque purification. Insertion at the correct locus and correct sequence was confirmed by PCR and sequencing.

### Plaque and comet assays

BSC-1 or NIH3T3 cells were seeded in six-well plates and grown to confluence. The virus strains were diluted in serum free DMEM (SFM) and approximately 25 PFU was added to each well. After incubation at 37°C in 5% CO_2_ for 1 h, the cells were washed twice and overlaid with either 1.5% carboxymethyl cellulose (CMC) in minimal essential medium (MEM) containing 2.5% FBS, 292 µg/ml L-glutamine, 100 units/ml penicillin and 100 µg/mL streptomycin for plaque assays, or DMEM-FPSG for comet assays. For experiments with imatinib treatment, imatinib mesylate (ChemiTek) at a final concentration of 10 µM or 0.01% of the carrier, DMSO, was included in the overlay. Cells were incubated for 3 days and then the overlay removed and cells stained with 1% crystal violet in methanol for visualisation.

### Single-step growth curves

BSC-1 cells were seeded into 12-well dishes, and confluent monolayers were infected with either WR, A36^YdF^, A36^Y112F^ or A36^Y132F^ in triplicate at a multiplicity of infection (MOI) of 5 for 1 h. The inoculum was removed, and the cell monolayer was washed three times with PBS. Cells and supernatant were collected at various times post-infection (4, 8, 12 and 24 h), freeze-thawed three times and the virus concentration determined by plaque assay of 10-fold serial dilutions in triplicate on BSC-1 cells. Cells were incubated for 3 days and then the overlay removed and cells stained with 1% crystal violet in methanol for visualisation.

### EEV assay

12-well dishes were seeded with BSC-1 or NIH3T3 cells and incubated with VACV strains (in triplicate) at an MOI of 0.1 for 1 h. Cells were then washed twice with PBS and DMEM-FPSG was added. For inhibitor treatments, imatinib mesylate at a final concentration of 10 µM, Cytochalasin D (Sigma-Aldrich) at a final concentration of 0.1 µg/ml or 0.01% of the carrier, DMSO, was added to the overlay media. The supernatants were collected at 16 hpi. To quantify the infectious EEV, plaque assays of 10-fold serial dilutions of the supernatant were performed on BSC-1 cells as described above. After 3 days cells were stained with methanol/1% crystal violet and plaques enumerated. All EEV assays were performed on at least three separate occasions.

### Electron microscopy

Confluent monolayers of BSC-1 cells were infected at an MOI of 5 and processed for TEM at 9 hpi. Briefly, cells were rinsed twice with PBS and fixed with 1.5% glutaraldehyde in Na-cacodylate buffer (0.1 M and 0.1 M sucrose) at pH 7.4 for 1 h. The samples were subsequently washed with PBS and postfixed with 1% osmium tetroxide in 0.1 M Na-cacodylate at pH 7.4 for 1 h. The cells were next dehydrated in a graded ethanol series. After dehydration, samples were embedded in Epon and after hardening of the embedding medium, the plastic of the multiwell dishes was removed using liquid nitrogen. Sections of 120 nm under various angles were cut with a diamond knife, stained first with uranyl acetate, subsequently with lead citrate, and examined in a Jeol 2100 (Jeol, Tokyo, Japan) at 200 kV.

### Fluorescent microscopy

BSC-1 cells were grown to 80% confluency on glass coverslips and infected with WR, A36^Y112^ and A36^YdF^ and fixed 8 hpi with 3% paraformaldehyde (PFA) in cytoskeletal buffer (CB) [10 mM 2-(N-morpholino) ethanesulfonic acid (MES) buffer, 0.15 M NaCl, 5 mM ethylene glycol tetraacetic acid (EGTA), 5 mM MgCl2, 50 mM glucose, pH 6.1] for 10 minutes at room temperature. Cells were blocked in blocking buffer (1% BSA, 2% FBS in CB) for 20 minutes then incubated for 40 minutes with 19C2 rat anti-B5 primary antibody (1/300) [80]. After three washes with PBS cells were incubated with AlexaFluor568 (Invitrogen) anti-rat secondary antibody (1/200) and AlexaFluor488 Phalloidin for 20 minutes. The coverslips were mounted on a glass slide with 0.3–1% (w/v) P-phenylenediamine (PPD; Sigma-Aldrich) in mowiol mounting media [10% (w/v) Polyvinyl Alcohol 4–88 (Sigma-Aldrich), 25% (w/v) glycerol, 0.1 M Tris, pH 8.5] and imaged with an Olympus microscope BX51 with filter sets 31001, 31002 and 31013v2 (Chroma). The resulting images analysed with Photoshop CS3 (Adobe).

For 3D-SIM, BSC-1 cells grown to 80% confluency on glass coverslips were infected with the fluorescently-tagged viruses at an MOI of 1. Transfection with plasmids expressing Lifeact-cerulean [79] was performed using Lipofectamine (Invitrogen), as described by the manufacturer. Cells were fixed at 8 hpi with 3% PFA for 10 min at RT then washed with PBS. If required, cells were blocked with blocking buffer and then probed with 19C2 rat anti-B5 primary antibody (1/300) and AlexaFluor568 anti-rat secondary antibody (1/200), diluted in the blocking buffer. The coverslips were mounted on a glass slide with 0.3–1% PPD in mowiol mounting media. Imaging was performed using a DeltaVision OMX 3D Structured Illumination Microscopy System (OMX 3D-SIM, Applied Precision Inc., Issaquah, USA), as described previously [81], [82]. General image handling was undertaken with either Image J or Adobe Photoshop CS4.

### Confocal microscopy

HeLa cells at 80% confluency were grown on Ibidi glass bottom μ-Dishes coated in fibronectin (5 µg/cm^2^, Sigma-Aldrich). Cells were infected with B5-YFP, A36^YdF^/B5-YFP or A36^Y112F^/B5-YFP and transfected with pE/L Lifeact-mRFP (Lipofectamine-2000, Invitrogen) [79]. Immediately prior to imaging on Olympus FV1000 confocal microscope at 6–9 hours post infection, media was changed to Lebovitz's L-15 (Invitrogen) with 5% FBS. Cells showing distinct viral B5-YFP localisations and the appearance of defined actin structures were imaged for 60 frames over 2 mins. Images were analysed with Olympus Fluoview Viewer (Ver. 03.1) and compiled with Adobe Photoshop CS4.

### Analyses and statistics

Plaque diameters were measured using Image J. Differences in EEV and plaque diameters were calculated using an unpaired t-test with Prism 5.0 (Graph Pad Software) software. To determine A36 distribution in 3D-SIM images, for each virus type 100 virus particles selected from five representative cells were classified as having polarised (P, less than 20% of virus circumference), intermediate (I, 20–80% of virus circumference) or circular (C, greater than 80% of virus circumference) A36 distribution (see Figure 6A). Classification was performed double-blinded.

## Supporting Information

Video S1 Live cell microscopy of VACV B5-YFP-induced actin nucleation. HeLa cells were infected with B5R-YFP (green), transfected with pE/L Lifeact-mRFP (red) and imaged for 80 seconds at 6–9 hours post infection. A virus particle is seen colocalising with F-actin tail of approximately 3–4 µm. See Figure 3D for still frames and representative scale bar.
(MOV)

Video S2 Live cell microscopy of VACV A36^Y112F^/B5-YFP-induced actin nucleation. HeLa cells were infected with A36^Y112F^/B5-YFP (green), transfected with pE/L Lifeact-mRFP (red) and imaged for 80 seconds at 6–9 hours post infection. A virus particle is seen undergoing rapid, presumably microtubule-dependent motility, before pausing and nucleating F-actin. See Figure 3D for still frames and representative scale bar.
(MOV)

Video S3 Live cell microscopy of VACV A36^YdF^/B5-YFP that appears in one frame to colocalise with F-actin. HeLa cells were infected with A36^YdF^/B5-YFP (green), transfected with pE/L Lifeact-mRFP (red) and imaged for 80 seconds at 6–9 hours post infection. Colocalisation between a virus particle and F-actin is observed transiently at the beginning of the movie but is not associated with virus motility. See Figure 3D for still frames and representative scale bar.
(MOV)
